# Palmitoylethanolamide-Incorporated Elastic Nano-Liposomes for Enhanced Transdermal Delivery and Anti-Inflammation

**DOI:** 10.3390/pharmaceutics16070876

**Published:** 2024-06-29

**Authors:** Chuanpeng Ren, Yanyun Ma, Yizhen Wang, Dan Luo, Yanhan Hong, Xinyuan Zhang, Hexiang Mei, Wei Liu

**Affiliations:** 1 The Institute of Biocelline Precision Dermatology, Shanghai 200031, China; yz.wang@biocelline.com (Y.W.); steve.mei@biocelline.com (H.M.); 2 Human Phenome Institute, Fudan University, Shanghai 201210, China; yanyunma@fudan.edu.cn; 3 Institute for Six-Sector Economy, Fudan University, Shanghai 201203, China; 4 Wuhan Bestcarrier Biotechnology Co., Ltd., Wuhan 430075, China; laurel565118@163.com (D.L.); hongyanhanhyh@126.com (Y.H.); 5 Shanghai Skinshield Clinical Testing and Technological Research Ltd., Shanghai 201210, China; 2019120467@stu.cmu.edu.cn; 6 National Engineering Research Center for Nanomedicine, Huazhong University of Science and Technology, Wuhan 430074, China

**Keywords:** palmitoylethanolamide, elastic nano-liposomes, transdermal delivery, anti-nociceptive, anti-inflammatory, transient receptor potential vanilloid 1

## Abstract

Palmitoylethanolamide (PEA) exhibits multiple skincare functions such as anti-nociceptive and anti-inflammatory effects. However, its topical application is limited due to its difficulty in bypassing the *stratum corneum* barrier, relatively low bioavailability, and low stability. Herein, elastic nano-liposomes (ENLs) with excellent deformability and elasticity were utilized as a novel drug delivery system to encapsulate PEA to overcome the abovementioned issues and enhance the biological effects on the skin. ENL was prepared with phosphatidylcholine, cholesterol, and cetyl-PG hydroxyethyl palmitamide with a molar ratio mimicking skin epidermal lipids, and PEA was loaded. The PEA-loaded ENL (PEA-ENL) demonstrated efficient transdermal delivery and enhanced skin retention, with negligible cytotoxicity toward HaCaT cells and no allergic reaction in the human skin patch test. Notably, PEA-ENL treatment increased cell migration and induced significant regulation in the expression of genes associated with anti-nociceptive, anti-inflammatory, and skin barrier repair. The mechanism of the anti-nociceptive and anti-inflammatory effects of PEA was further investigated and explained by molecular docking site analysis. This novel PEA-ENL, with efficient transdermal delivery efficiency and multiple skincare functionalities, is promising for topical application.

## 1. Introduction

Palmitoylethanolamide (PEA), also known as paltitamide MEA, is a lipid amide that widely exists in mammalian cells and tissues [1]. PEA belongs to the *N*-acylethanolamine (NAE) fatty acid amide family and has a hydrophobic property with a partition coefficient (LogP) of 5.74 [2]. As a nutraceutical, several preclinical and clinical studies have suggested that PEA has pleiotropic clinical benefits including anti-inflammatory, antimicrobial, analgesic, antipyretic, and neuroprotective effects [3,4,5,6]. These therapeutic effects can be achieved due to PEA mechanisms that affect multiple pathways including targeting the nuclear receptor peroxisome proliferator-achieved alpha (PPARα), G protein-coupled receptor 55 (GPR55), and G protein-coupled receptor 119 (GPR119) [4,7]. As an endocannabinoid (eCB)-like lipid mediator [8], PEA can indirectly activate cannabinoid receptors 1 and 2 (CB1 and CB2) by inhibiting the degradation of anandamide (AEA) [2]. Moreover, some studies have demonstrated that PEA can target transient receptor potential vanilloid 1 (TRPV1) through anti-nociceptive effects [1,9]. In addition, PEA can down-regulate mast cells in peripheral tissues and the central nervous system [10,11]; thus, it shows extraordinary performance in immunity with antiallergic activities [9].

The majority of PEA medications launched on the market are for oral administration, such as Normast^®^, Pelvilen^®^, and PeaPure^®^ for human application and Redonyl^®^ Ultra for veterinary use [12]. Nowadays, the anti-nociceptive and anti-inflammatory functions of PEA have attracted attention for topical application. Physiogel AI^®^, a cream containing PEA, has been marketed for clinically treating skin dryness and irritation [13]. Although PEA has demonstrated excellent therapeutic effects, its bioavailability is remarkably constrained due to its poor water solubility and limited stability. Nano-drug delivery systems provide a solution to solve these problems. A nano-sized delivery system could effectively enhance transdermal delivery via skin surface adhesion and *stratum corneum* penetration. Furthermore, the incorporation of active pharmaceutical ingredients (APIs) into the delivery system could increase water solubility, enhance stability, and in the meantime decrease the occurrence of undesired side effects [14]. A variety of drug delivery systems including nanostructured lipid carriers (NLCs), solid lipid nanoparticles (SLNs), liposomes, niosomes, nanocrystals, and nanospheres have been successfully developed for dermal and transdermal drug delivery [14,15,16]. Although the abovementioned delivery systems have been widely utilized for topical application, their skin permeation efficiency is relatively low [17]. As a novel transdermal delivery system composed of phospholipids and also surfactants and elastic nano-liposomes (ENLs) can not only encapsulate both hydrophilic and hydrophobic drugs but also show flexibility and elasticity in the lipid structure. Surfactants can impart flexibility in structure, and the ultra-deformability of these vesicles facilitates deeper skin penetration than conventional ones [18,19]. A recent study demonstrated the efficacy of ENL-containing tocopherol acetate, showing superior transdermal permeation both in vitro and ex vivo [20]. This enhanced permeation is attributed to the ENLs’ ability to squeeze through intercellular spaces within the skin barrier, effectively delivering active compounds to targeted sites [20]. However, due to the presence of surfactants, skin barrier function may be hampered during the penetration of ENLs.

Herein, we developed a novel ENL with a formulation mimicking the *stratum corneum* lipids ratio to enhance *stratum corneum* penetration efficiency and in the meantime maintain skin barrier functionality. PEA, with anti-nociceptive and anti-inflammatory effects, was further loaded into the ENL to develop a PEA-loaded ENL (PEA-ENL) to achieve multiple skincare functions. Basic *stratum corneum* lipids, that is, ceremide, cholesterol, and phosphatidylcholine, with a molar ratio similar to skin biological lipids were utilized for ENL preparation. Through ENL encapsulation, the solubility and stability of PEA were improved, which further led to enhanced bioavailability. Furthermore, taking advantage of the nano-sized vesicle with high deformability and elasticity [21,22,23], the PEA-ENL exhibited efficient transdermal delivery and increased skin retention. In vitro pharmacological effects induced by the PEA-ENL in human keratinocyte (HaCaT) cells as well as the human skin patch test were carried out to evaluate its safety. Gene and protein expression and molecular docking site analyses were performed to illustrate its mechanism of skincare function. This PEA-loaded ENL with anti-nociceptive and anti-inflammatory effects shows potential for skincare application.

## 2. Materials and Methods

### 2.1. Materials

Hydrogenated lecithin and polyethylene glycol (PEG)-40 hydrogenated castor oil (CO40) were obtained from Sungwoo Chemical, Tokyo, Japan. Cholesterol was obtained from Sinopharm, Beijing, China. Pentanediol was purchased from B&B, Seoul, Republic of Korea. Trioctyl/capric glyceride and stearyl polyether-21 were purchased from Croda, Yorkshire, UK. Fetal bovine serum (FBS), Dulbecco’s modified Eagle medium (DMEM), phosphate-buffered saline (PBS, pH 7.4), penicillin, streptomycin, trypsin-EDTA, and dimethyl sulfoxide (DMSO) were purchased from Gibco, New York, NY, USA. Rhodamine B isothiocyanate (RhoB) was purchased from Sigma Aldrich, St. Louis, MO, USA. Fluo-4 AM was acquired from Yeasen Biotech Co., Ltd., Shanghai, China. The CCK-8 assay kit was purchased from APE×BIO, Houston, TX, USA.

### 2.2. Preparation and Characterization of the PEA-ENL

The PEA-ENL was prepared using a high-pressure homogenization technique. Briefly, 0.127% (*w/w*) hydrogenated lecithin, 0.065% (*w/w*) cholesterol, 0.1% (*w/w*) cetyl-PG hydroxyethyl palmitamide, 1% (*w/w*) PEA, 20% (*w/w*) pentanediol, and 3% (*w/w*) caprylic decanoic acid triglyceride were mixed and stirred at 60 °C until fully soluble to produce phase A. Next, 2% (*w/w*) CO40 and 4% (*w/w*) stearyl polyether-21 were mixed together to produce phase B. After mixing phase B with phase A and stirring at 60 °C to ensure full dissolution, distilled water was added to phase AB, stirred at the same temperature until totally dissolved, and then homogenized at 800 bar using an AMH-3 micro-spray high-pressure homogenizer (Antos Nanotechnology, Suzhou, China) to prepare the PEA-ENL. The process of homogenization was repeated 3 times in order to produce nano-size particles and disperse them more evenly. Finally, purification was performed via ultrafiltration (MWCO 3.5 kDa, Amicon Ultra, Millipore, Billerica, MA, USA) at 4000 rpm for 30 min to remove the unencapsulated PEA. An RhoB-loaded ENL (RhoB-ENL) was prepared following the same method described above, except that RhoB was added to phase A to replace PEA.

The particle size and polydispersity index (PDI) of the PEA-ENL were measured using dynamic light scattering (DLS) using a Zetasizer/Nano-ZS90 instrument (Malvern Instruments, Malvern, UK). The morphology of the PEA-ENL was observed using transmission electron microscopy (TEM, HT7700, Hitachi, Tokyo, Japan). The PEA-ENL was diluted 400 times with deionized water, dropped on the copper grid, stained with 1% phosphomolybdic acid, and air dried before TEM observation.

The content of PEA was determined using high-performance liquid chromatography (HPLC) (Shimadzu Instruments, Columbia, MD, USA) with a Sepax Bio-C18 column (4.6 mm × 250 mm, 5.0 µm, 300 Å, Suzhou, China) at a UV wavelength of 214 nm, 30 °C. Methanol/ultrapure water = 94/6 (*v/v*) was applied as the mobile phase. The retention time of PEA was about 5.1 min, the flow rate was 1.0 mL/min, and the injection volume was kept at 10 μL. Ultrafiltration centrifugation was applied to remove the unencapsulated PEA, and the encapsulation efficiency (EE) and drug-loading efficiency (DL) of the PEA-ENL were calculated using the following equations:

(1) $$DL(\%)=\frac{We}{We+Wm}\times 100$$

(2) $$EE(\%)=\frac{We}{We+Wf}\times$$

In which We is the mass of the active ingredients encapsulated in the nanocarrier, Wm is the total mass of the nanocarrier, and Wf is the mass of the free active ingredients not encapsulated in the nanocarrier.

### 2.3. In Vitro Skin Retention

Skin tissue was taken from the back of BAMA miniature pigs (5–6 kg body weight) with undamaged hair follicles, purchased from Zhifu Yurong Biological Studio (Yantai, Shandong, China). The transdermal properties of the PEA-ENL were investigated using the Franz diffusion cell method. Briefly, the BAMA pigskin was fixed between the supply pool and the receiving pool, with the *stratum corneum* oriented toward the supply pool. Next, 0.5 mL of the PEA-ENL and 0.5 mL free PEA containing the same concentration of PEA, were added to the supply pool and then uniformly applied to the BAMA pigskin.

The Franz diffusion cells were maintained at 37 °C using a recirculating water bath and the PBS buffer (pH 7.4) in the receptor chambers was stirred continuously at 300 rpm. After 24 h, the skin was removed and ground with 2 mL methanol, ultrasonicated for 10 min, and then centrifuged. The supernatant was filtered and taken for HPLC analysis to calculate the retention of PEA per unit area of skin.

### 2.4. Fluorescence Microscopy Observation of Skin Penetration

To visualize the transdermal permeation process, 0.5 mL of the RhoB-ENL or free RhoB solution with the same RhoB concentration was added to the supply pools and applied uniformly to the BAMA pigskin. During percutaneous permeation, the skin was removed at different time points (2 h, 4 h, and 8 h), and skin tissue sections were prepared using a cryotome (Thermo Scientific, HM525NX, Shanghai, China). The distribution of RhoB in the skin tissue sections was observed using fluorescence microscopy (IX71, Olympus, Tokyo, Japan).

### 2.5. Human Skin Patch Test

In the human skin occlusive patch test, 16 male participants and 16 female volunteers within the age range of 25 to 45 years old were randomly divided into two groups as the PEA-ENL-treated group and the control group. Next, 0.020 to 0.025 g cream with or without PEA-ENL was loaded into patches (7 mm × 7 mm), applied onto the forearm of the volunteers with light palm pressure, and protected by a self-sticking tape. After contacting the skin surface (about 49 mm^2^ area) for 24 h, the patches were removed, and the residual sample was cleaned from the skin. Skin irritative reactions were evaluated 0.5, 24, and 48 h after patch removal. The skin reaction scores were recorded according to the Safety and Technical Standards for Cosmetics (2015).

### 2.6. Cell Culture for HaCaT and HSF Cells

The human keratinocyte cells (HaCaT, Kunming Cell Bank of Chinese Academy of Sciences) and human skin fibroblast cells (HSF, Kunming Cell Bank of Chinese Academy of Sciences) were cultured in DMEM supplemented with 10% FBS and 1% penicillin/streptomycin. The cells were incubated in a humidified atmosphere with 5% CO_2_ at 37 °C. The HaCaT and HSF cells were cultured in Dulbecco’s modified Eagle’s medium (DMEM) supplemented with 10% FBS. All cells were cultured at 37 °C in a 5% CO_2_ humidified incubator. Cells treated with PEA-ENL were harvested for further analysis.

### 2.7. Cell Viability Assays

Cell viability was assessed using CCK-8 assay. Briefly, HaCaT cells (1 × 10^4^ cells/well) were cultured in 96-well plates for 24 h. After removing the culture medium, different concentrations of PEA-ENL and capsaicin were incubated for 24 h, followed by the addition of 10 μL of CCK-8 buffer to each well and incubation at 37 °C for another 2 h. Then, the absorbance at 450 nm was measured using a microplate reader (Tecan Spark, Männedorf, Switzerland).

### 2.8. Cell Migration Assays

HaCaT and HSF cells were seeded separately in 6-well plates at a density of 1 × 10^6^ or 4 × 10^5^ cells per well, respectively. After being cultured for 24 h at 37 °C, straight lines were scratched on the cells in each well using 200 μL sterile pipette tips, followed by washing three times with sterile PBS to remove cell debris. Next, the HaCaT and HSF cells were treated with the free PEA or PEA-ENL at an equal PEA concentration of 10 μM. The empty ENL was administered as a control. Photographs of the wounded area were taken under the optical microscope (MshOt, MF52-N, Guangzhou, China) at the start of treatment (0 h) and 24 h after treatment.

### 2.9. RNA Isolation and Quantitative Real-Time PCR (qRT-PCR)

Total RNA was isolated from cells using an HP Total RNA Kit (Omega Biotech, Stamford, CT, USA) according to the manufacturer’s protocol. Synthesis of cDNA with reverse transcriptase was performed using an M-MLV First Strand Kit (Life Technologies, Gaithersburg, MD, USA). Real-time qRT-PCR was performed on an ABI Prism 7900 Real-Time PCR system (Applied Biosystems, Foster City, CA, USA) using the SYBR-Green-based method. Relative expression levels of mRNA were determined after normalization to Glyceraldehyde 3-phosphate dehydrogenase (GAPDH).

### 2.10. Calcium Transient Assay

HaCaT cells were cultured in 6-well culture plates for 24 h. After removing the culture medium, 1 μM of capsaicin and 10 μM of the PEA-ENL were added into the culture plates and incubated for 24 h. The cells were then stained with Fluo-4 AM at 37 °C for 1 h. After incubation, the cells were washed twice with PBS and photographed using fluorescence microscope (Leica DMI4000, Wetzlar, Germany). The fluorophore was excited at 488 nm, while emission at 530 nm was acquired.

### 2.11. Molecular Docking Sites Analysis

The structure of PEA was modeled with GaussianView [24] and then optimized with M062X [25,26,27]/LANL2DZ [28] in Gaussian 16 [29]. The optimized structure was further imported into AutodockTools 1.5.6. The protein structures of CB1 (PDB ID: 5TGZ) and TRPV1 (PDB ID: 7LPB) were downloaded from the RCSB Protein Data Bank (PDB). Subsequently, molecular docking was performed with AutoDock 4.2.6 [30,31] software package by using a built-in semi-empirical free energy scoring function. Specific interacting mode analysis was performed with PyMol 2.3.0.

### 2.12. Statistics

All results are shown as the mean and standard deviation from at least three independent experiments. An independent two groups test or one-way analysis of variance (ANOVA) with Fisher’s least significant difference (LSD) multiple comparison test and two-tailed Student’s *t*-test was employed for statistical analysis. A *p*-value of less than 0.05 was considered significant.

## 3. Results

### 3.1. Characteristics of the PEA-ENL

**Figure 1:**
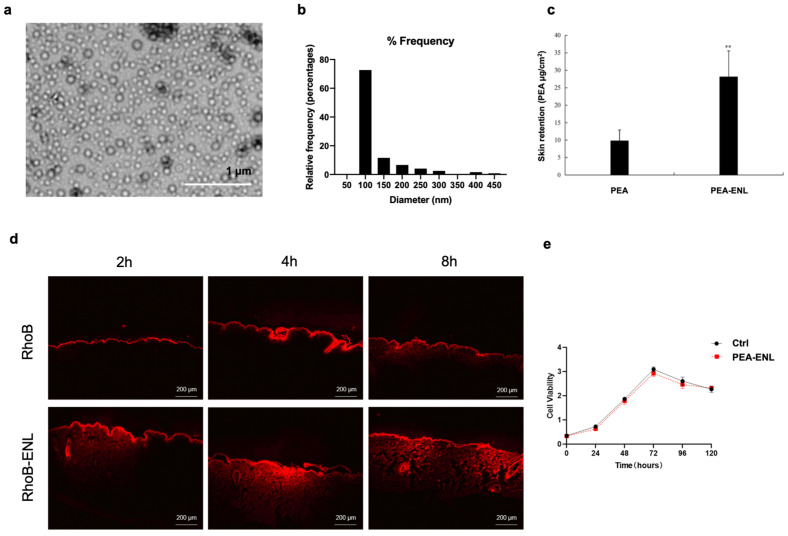
Characteristics of the PEA-ENL: (**a**) TEM image of the PEA-ENL; (**b**) size distribution histogram of the PEA-ENL; (**c**) PEA skin retention after treating porcine skin with the free PEA and PEA-ENL for 24 h. (**d**) fluorescence microscope images of porcine skin treated with the free RhoB or RhoB-ENL with equal RhoB concentrations for up to 8 h; (**e**) cell viability of HaCaT cells treated with the PEA-ENL (contained 10 µM PEA). Data are presented as the mean ± SD, *n* = 3, **: *p* < 0.01, Student’s *t*-test.

The hydrodynamic size of the optimized PEA-ENL prepared using a high-pressure homogenization technique was 155.8 ± 14.5 nm, measured using DLS (Figure S1). TEM was utilized to visualize the morphology of the PEA-ENL and characterize its diameter. As seen in Figure 1a, a uniformly distributed spherical shape was observed, suggesting the successful preparation of the PEA-ENL using the high-pressure homogenization technique. The size distribution histogram of the PEA-ENL was analyzed based on the TEM image. The average particle size measured using TEM (Figure 1b) was slightly smaller than the hydrodynamic size measured using DLS (Figure S1): 72.7% of the PEA-ENLs were within the size range of 50 nm to 100 nm, while 11.5% of the PEA-ENLs were within the size range of 100 nm to 150 nm. The size distribution results demonstrated that the majority of the PEA-ENL displayed suitable size for skin surface adhesion and *stratum corneum* permeation [32]. The encapsulation efficiency and drug-loading efficiency of the optimized PEA-ENL were 94.3 ± 0.8% and 1.0 ± 0.4%, respectively. The transdermal delivery properties of the PEA-ENL were determined via skin retention analysis and skin permeation process visualization. Figure 1c shows that after 24 h of topical application, the amount of PEA retained in the porcine skin in the PEA-ENL group was 2.8-fold higher than the group treated with free PEA. To further investigate the effect of the ENL in enhancing PEA transdermal penetration, an in vitro transdermal diffusion test was conducted. A fluorescent dye, rhodamine B isothiocyanate (RhoB), was loaded into the ENL as a tracer molecule. The transdermal delivery process was visualized through the use of fluorescence microscopy after topical application at different time intervals. As shown in Figure 1d, the red fluorescent signal in the group treated with the RhoB-ENL was remarkably stronger than that in the control (Ctrl) group treated with free RhoB at the same RhoB concentration, indicating that ENLs could effectively promote the transdermal delivery and penetration of encapsulated cargos to the deeper layer of the skin. The integrated density of the fluorescent signal was further quantified using ImageJ (V. 1.44p, NIH, USA) and presented in Table S1.

To eliminate the safety concern of the PEA-ENL, in vitro cell viability and potential induced allergic reactions in humans were further investigated. HaCaT cells were used to assess the toxicity of the PEA-ENL containing 10 µM PEA. As seen in Figure 1e, compared with the Ctrl group, no obvious cytotoxicity was observed in the HaCaT cells treated with the PEA-ENL for 5 days. Furthermore, to evaluate its potential allergic reaction-inducing effect in humans, a skin patch test was performed. A total of 32 volunteers, with an equal number of males and females, were randomly treated with patches containing 0.020 to 0.025 g cream with or without the PEA-ENL for 24 h. The skin reaction level after removing the patches for 0.5, 24, and 48 h was observed and recorded as different scores. As seen in Table 1, no allergic reaction was noticed in either participant in the PEA-ENL-treated group or the Ctrl groups, demonstrating the safety of the PEA-ENL.

Taken together, these data suggest that the optimized ENL with an average diameter of 155.8 nm can serve as an ideal carrier to effectively encapsulate PEA and promote its transdermal delivery with negligible cytotoxicity in HaCaT cells and insignificant allergic reactions on human skin.

### 3.2. Effects of the PEA-ENL on Cell Migration

**Figure 2:**
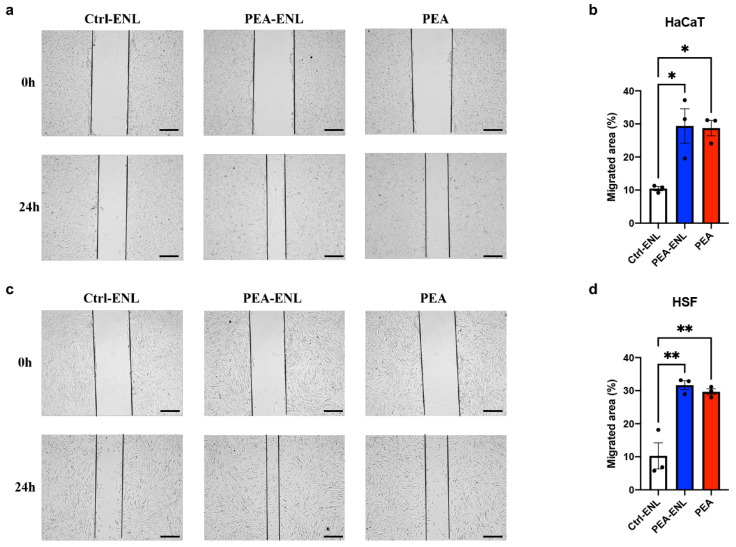
Cell migration assays: (**a**) microscopic images of HaCaT cells at the start of treatment (0 h) and after 24 h of treatment with the Ctrl-ENL, PEA-ENL, and PEA (2 µg/mL); (**b**) quantification of the migrated area of HaCaT after 24 h of treatment with the Ctrl-ENL, PEA-ENL, and PEA; (**c**) microscopic images of HSF at the start of the treatment (0 h) and after 24 h of treatment with the Ctrl-ENL, PEA-ENL, and PEA (2 µg/mL); (**d**) quantification of the migrated area of HSF after 24 h of treatment with the Ctrl-ENL, PEA-ENL, and PEA. *n* = 3. Data are presented as mean ± SD. *: *p* < 0.05, **: *p* < 0.01; ANOVA; scale bar 200 μm.

To evaluate the effects of the PEA-ENL in promoting skin repair, transwell cell migration assays were conducted using two human skin cell lines, HaCaT and HSF. The cells were treated with PEA (2 µg/mL), the PEA-ENL containing the equivalent amount of PEA, and the empty ENL (Ctrl-ENL). As shown in Figure 2a, at the initial time, all of the groups displayed identical wound distances. After 24 h treatment, the wound distance was remarkably reduced in both the PEA and PEA-ENL groups compared to the Ctrl-ENL group. The migrated area is further quantified in Figure 2b. Compared to the Ctrl-ENL group, the migrated area of HaCaT cells treated with PEA and the PEA-ENL changed by 2.7 ± 0.3-fold (* *p* < 0.05) and 2.8 ± 0.3-fold (* *p* < 0.05), respectively. As shown in Figure 2c,d, the treatment of HSF cells with the PEA-ENL and PEA presented a greater migrated area than the Ctrl-ENL group with 4.0 ± 1.3-fold (** *p* < 0.01) and 3.8 ± 1.1-fold (** *p* < 0.01) increases, respectively. Notably, the differences between the PEA and PEA-ENL groups in both cell lines were not significant, suggesting that the encapsulated PEA in the ENL delivery system suitably maintained its cell proliferation functionality. These results suggest that 24 h treatment with the PEA-ENL can effectively promote cell migration in both HaCaT and HSF cells.

### 3.3. Multi-Targets Effects of the PEA-ENL in HaCaT Cells

**Figure 3:**
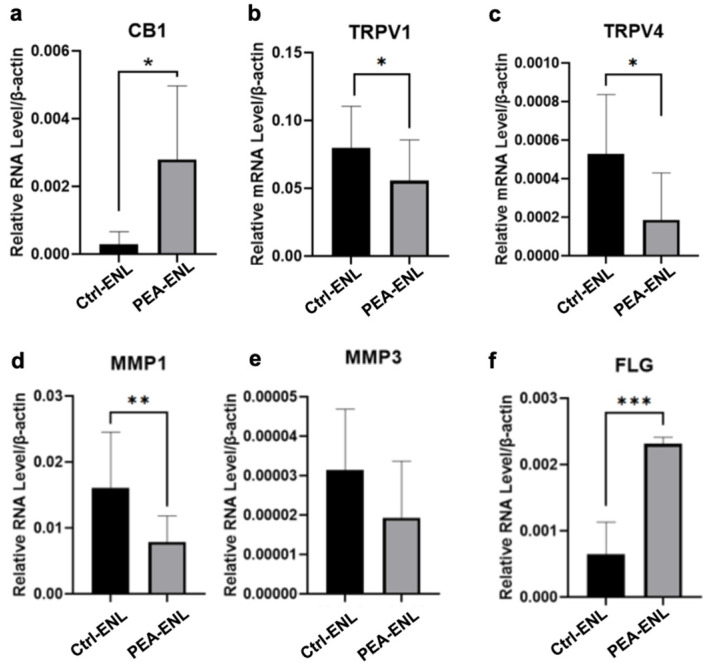
Gene expressions in HaCaT cells after treatment: (**a**) *CB1* expression in HaCaT cells after 12 h treatment with the Ctrl-ENL and PEA-ENL; (**b**) *TRPV1* expression in HaCaT cells after treatment; (**c**) *TRPV4* expression in HaCaT cells after treatment; (**d**) *MMP1* expression in HaCaT cells after treatment; (**e**) *MMP3* expression in HaCaT cells after treatment; (**f**) *FLG* expression in HaCaT cells after treatment. *n* = 3. Data are presented as the mean SD. *: *p* < 0.05, **: *p* < 0.01, ***: *p* < 0.001; Student’s *t*-test.

To investigate the effects of the PEA-ENL in HaCaT cells, the expression of *CB1*, *TRPV1*, and *TRPV4* was analyzed. The expression of *CB1* in HaCaT cells following 12 h treatment with the PEA-ENL increased significantly by five-fold (Figure 3a), while a significant reduction in the expression of *TRPV1* (Figure 3b) and *TRPV4* (Figure 3c) was observed after treatment with the PEA-ENL compared with the Ctrl group. These data suggest that the administration of the PEA-ENL demonstrated notable anti-nociceptive properties in HaCaT cells.

Interestingly, compared with the Ctrl group, the expression of matrix metallopeptidases including *MMP1* (Figure 3d) and *MMP3* (Figure 3e) was downregulated following treatment with the PEA-ENL, indicating that treatment with the PEA-ENL effectively inhibits collagen degradation. The important role of filaggrin (FLG) in skin barrier function and maintaining skin hydration has been demonstrated by the strong association between the loss of function of FLG and atopic dermatitis [33]. In this study, we further assessed the effects of the PEA-ENL on the expression of *FLG* in HaCaT cells. The expression of *FLG* in HaCaT was three-fold upregulated following treatment with the PEA-ENL compared with the Ctrl-ENL-treated group (Figure 3f).

### 3.4. Competitive Binding of PEA at TRPV1 Channels in HaCaT Cells

**Figure 4:**
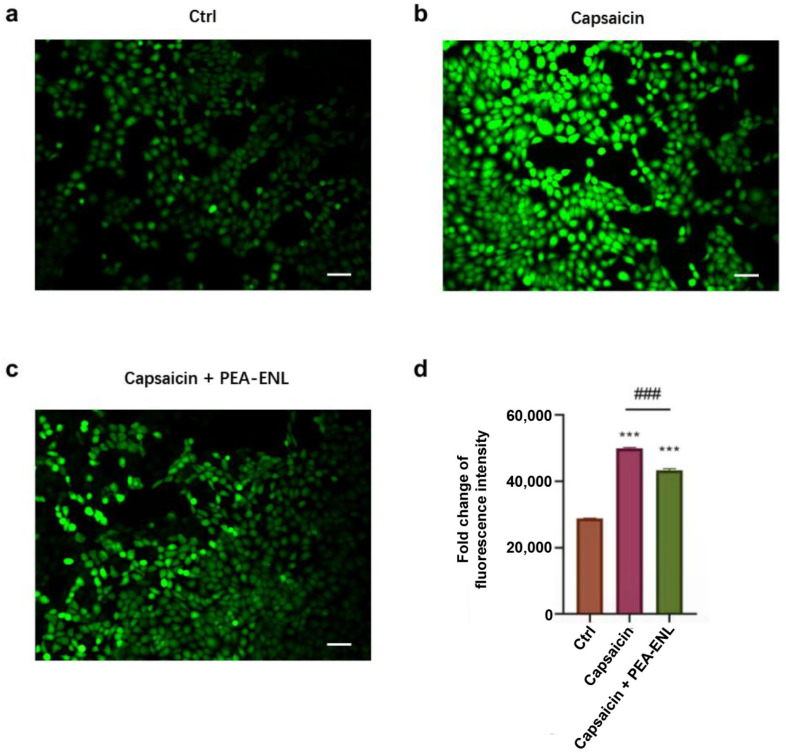
Effect of capsaicin (1 μM) and the PEA-ENL (10 μM) on intracellular Ca^2+^ flux. Confocal microscopy images of HaCaT cells after treatment with (**a**) Ctrl, (**b**) capsaicin (1 µM), and (**c**) the combination of capsaicin (1 μM) and the PEA-ENL (10 μM), respectively. (**d**) Quantitative analysis of fluorescence intensity in HaCaT cells after treatment. Data are presented as the mean ± SD, *n* = 3. Compared with the Ctrl, ***: *p* < 0.001; Compared with capsaicin, ^###^: *p* < 0.001, Student’s *t*-test; scale bar 100 μm.

Capsaicin, a TRPV1 agonist, was employed to assess TRPV1 functional expression. In this study, 1 μM of capsaicin was selected to induce calcium influx in HaCaT cells. The inhibition effects of the PEA-ENL on TRPV1 channels were investigated by assessing changes in intracellular free Ca^2+^ concentration. The accumulation of Ca^2+^ in HaCaT cells was visualized using Fluo-4 AM ester as a fluorophore. Compared to the Ctrl (Figure 4a), significantly enhanced fluorescence intensity was observed in the capsaicin-treated cells (Figure 4b). In contrast, decreased green fluorescence was noticed after the addition of a PEA-ENL containing 10 μM of PEA (Figure 4c). The fluorescence intensity fold change was further quantified. As shown in Figure 4d, the fluorescence intensity of capsaicin was 73.61% higher than the Ctrl, suggesting that capsaicin could significantly facilitate Ca^2+^ accumulation (*** *p* < 0.001). After the addition of the PEA-ENL, 13.28% decreased fluorescence intensity was observed compared to the cells treated with capsaicin only, indicating that PEA-ENL remarkably decreased Ca^2+^ accumulation (^###^: *p* < 0.001, Figure 4d).

**Figure 5:**
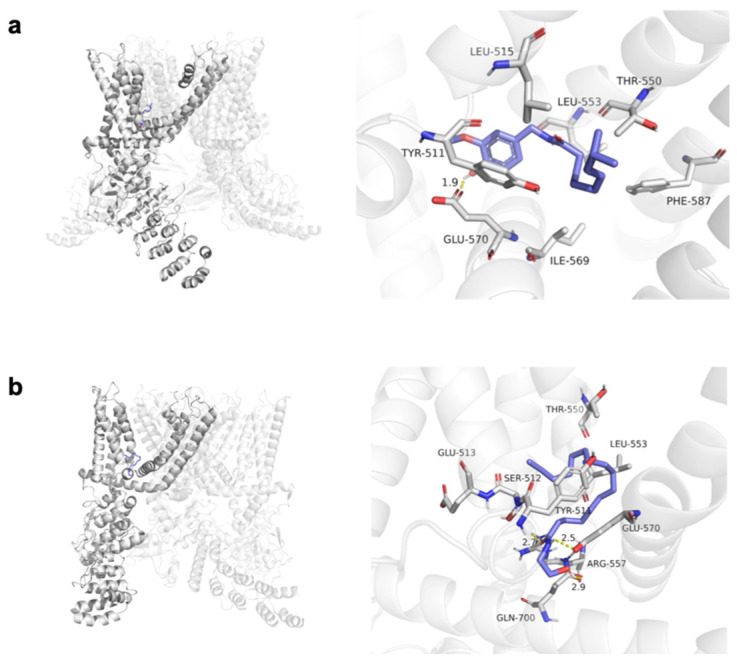
Molecular docking sites and poses of (**a**) capsaicin and (**b**) PEA for TRPV1 (PDB ID: 7LPB). The docking score of capsaicin and PEA binding with TRPV1 was −7.8 kcal/mol and −6.7 kcal/mol, respectively. The TRPV1 protein backbone is represented as a cartoon. The active site residues are shown in stick representation with carbon in grey, oxygen in red and nitrogen in cyan. The ligand molecule shown in stick representation in blue is docked into the binding cavity. Dotted lines represent the intermolecular H-bonds.

To further investigate the role of PEA in regulating TRPV1 in HaCaT cells, the molecular docking sites and docking score of both capsaicin and PEA with TRPV1 were analyzed via simulation using AutoDockTools. The molecular docking score of capsaicin (Figure 5a) and PEA (Figure 5b) for TRPV1 protein was −7.8 kcal/mol and −6.7 kcal/mol, respectively. As reported [34], the molecular docking results of the TRPV1 agonist capsaicin demonstrated that a strong hydrogen bond formed between capsaicin and residue Glu570 of TRPV1. However, in contrast with the most common TRPV1 agonists like capsaicin, the interaction between PEA and TRPV1 exhibited a quite different mode, mainly reflected in the residues that formed hydrogen bonds with the molecules. Besides Glu570, PEA also formed two extra hydrogen bonds with residues Arg557 and Gln700, and the latter one was located in the C-terminal region [35] of the protein (Figure 5b).

In conclusion, the calcium influx assay and molecular docking analyses revealed that PEA competitively interferes with capsaicin binding to TRPV1 channels. These results demonstrate that the PEA-ENL effectively inhibits intracellular Ca^2+^ levels in HaCaT cells by competitively binding to TRPV1 channels, thereby potentially exerting anti-nociceptive effects.

### 3.5. Anti-Inflammatory Effects of the PEA-ENL in HaCaT Cells

**Figure 6:**
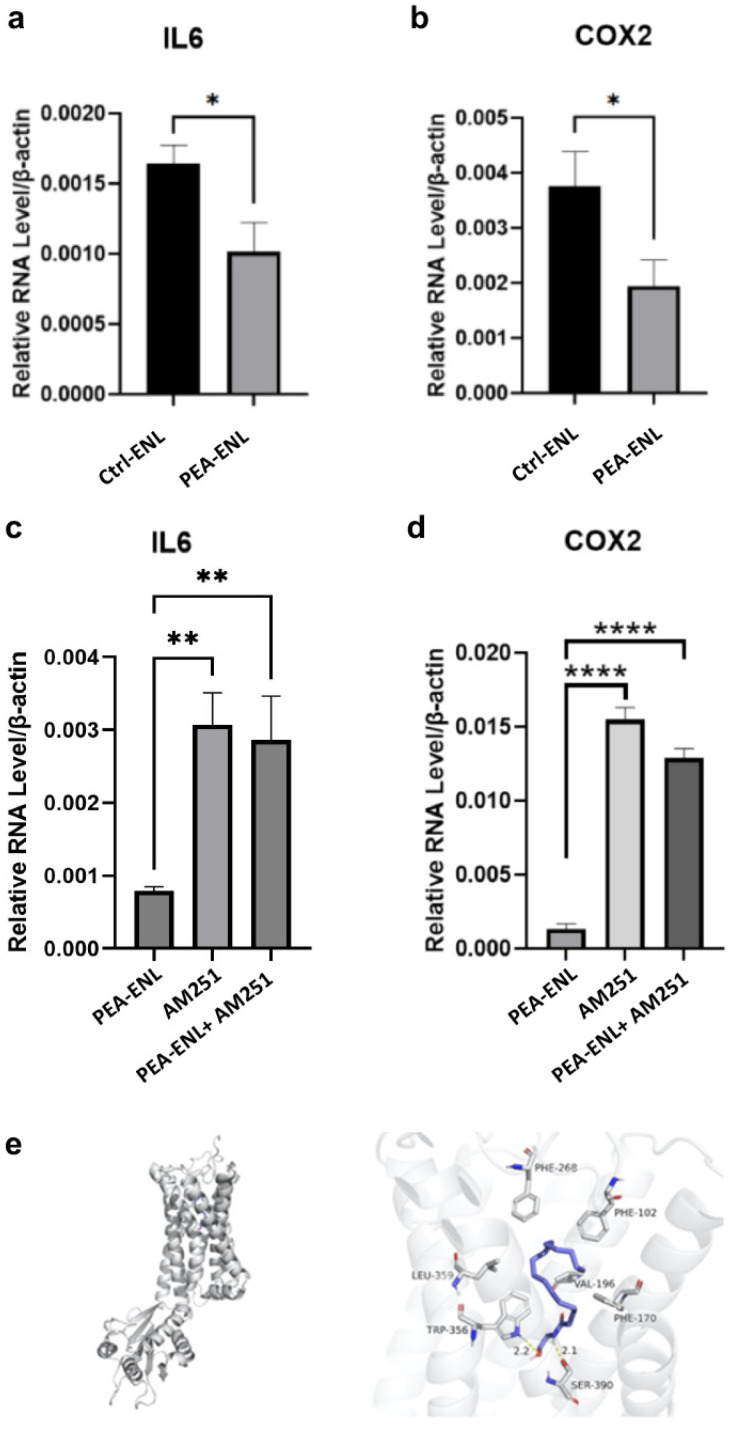
Inflammatory gene expressions in HaCaT cells after 12 h treatment. (**a**) *IL6* expression after treatment with the Ctrl-ENL and PEA-ENL, (**b**) *COX2* expression after treatment with Ctrl-ENL and PEA-ENL, (**c**) *IL6* expression after treatment with the PEA-ENL and AM251, (**d**) *COX2* expression after treatment with the PEA-ENL and AM251, and (**e**) molecular docking sites and poses of PEA for CB1 receptor (PDB ID: 5TGZ). The CB1 protein backbone is represented as a cartoon. The active site residues are shown in stick representation with carbon in grey, oxygen in red and nitrogen in cyan. PEA shown in stick representation in blue is docked into the binding cavity. Dotted lines represent the intermolecular H-bonds. Data are presented as the mean ± SD, *n* = 3. *: *p* < 0.05, **: *p* < 0.01, ****: *p* < 0.0001; Student’s *t*-test.

The anti-inflammatory effects of the PEA-ENL in HaCaT cells were evaluated by investigating the expression of the inflammatory cytokines interleukin 6 (*IL6*) and cyclooxygenase 2 (*COX2*). As shown in Figure 6a,b, HaCaT cells treated with the PEA-ENL (10 µM) showed a significant reduction in the expression of *IL6* and *COX2* compared with the Ctrl-ENL-treated group, indicating the anti-inflammatory effects of the PEA-ENL. To further investigate if the downregulated expression of *IL6* and *COX2* were induced by CB1 activation, a CB1 receptor-selective antagonist AM251 (10 µM) was used. The expression of *IL6* and *COX2* in HaCaT cells treated with the PEA-ENL (10 µM) was significantly enhanced following treatment with AM251 (Figure 6c,d). Therefore, these data indicate that the PEA-ENL displayed an effective anti-inflammatory effect by activating CB1.

AM251 exhibits a strong ability to bind with CB1. Through analysis of molecular docking, the binding score was as high as −10.1 kcal/mol (see Figure S2). As shown in Figure 6e, PEA was also precisely docked into the binding pocket of the CB1 receptor and formed strong hydrogen bonds with protein residues around the pocket. The docking score of PEA for the CB1 receptor was −7.3 kcal/mol (Figure 6e), suggesting that PEA released from the PEA-ENL is capable of binding to the CB1 receptor, consequently exhibiting anti-inflammatory effects. This also indicated that the application of AM251 blocked the effect of the PEA-ENL on CB1 in HaCaT cells.

## 4. Discussion

PEA exhibits potential anti-inflammatory and anti-nociceptive properties and is promising for topical application [7]. The main challenges to improving efficient skincare effectiveness are limited by low bioavailability, low stability, and the difficulty in overcoming the *stratum corneum* barrier. Therefore, a drug delivery system needs to be employed to enhance skin penetration and improve the stability of PEA. Compared with other widely used drug delivery systems such as solid lipid nanoparticles, nanostructured lipid carriers, liposomes, and nanocrystals, the ENL shows much higher vesicle deformability and thus could more efficiently penetrate the *stratum corneum* barrier [36]. Deformability is important and worth further exploration in our follow-up research work. In comparison with physical permeation enhancement methods (e.g., microneedles and electroporation), the use of an ENL is non-invasive. Hence, an ENL, a novel delivery carrier, was selected to load PEA to improve its bioavailability and stability, decrease its side effects, and enhance its transdermal delivery to achieve desirable skincare effects.

The size of the ENL plays a vital role in determining transdermal delivery efficiency. Liposomes with a size of 120 nm diameter have shown significantly enhanced dermal delivery of API into the skin compared with larger liposomes [37]. In this study, the PEA-ENL with an average size of 155.8 ± 14.5 nm diameter showed a better skin retention capability than the free PEA-treated group (Figure 1c), indicating that encapsulation of PEA into the ENL could not only improve the transdermal delivery of PEA but also show the potential to protect encapsulated PEA from oxidization and degradation on the skin surface after topical application. Moreover, the RhoB-loaded ENL with similar particle size exhibited a remarkably enhanced transdermal diffusion rate and deep skin penetration (Figure 1d), which further demonstrated the capabilities of the ENL as a novel carrier for transdermal delivery.

In order to explore the skin pharmacological effects of the PEA-ENL, two promising therapeutic targets, CB1 and TRPV1, were studied. A previous study demonstrated that the activation of CB1 can induce anti-nociceptive effects, which can be blocked by CB1 antagonists [38]. TRPV1, also known as the capsaicin receptor, belongs to a family of transient receptor potential (TRP) channels. The TRPV1 receptor can be activated by thermal, physical, and electrochemical stimuli and is responsible for skin burning and nociceptive sensation [34,35]. Regarding the in vitro experiments in HaCaT cells, an increase in the expression of *CB1* and a decrease in the expression of *TRPV1* was observed following treatment with the PEA-ENL (Figure 3a,b). These results indicate the anti-nociceptive effects of PEA-ENL treatment in HaCaT cells.

To further investigate the PEA-ENL’s functions as a TRPV1 antagonist, capsaicin-induced calcium influx was investigated and quantified. A wide variety of environmental stimuli can activate TRPV1, causing Ca^2+^ and Na^+^ influx from the extracellular space to cytosols. The pathophysiological relevance of TRPV1 with skin sensitivity has been investigated in several studies [35,36]. TRPV1 stimulation was able to delay barrier recovery, whereas the administration of a TRPV1 antagonist improves barrier repair. The efferent function after sensations of itch, pain, warmth, and chemical stimuli and the local release of neuropeptides were also related to TRPV1 activation [39]. Our results indicate the functionality of the PEA-ENL in blocking capsaicin-induced TRPV1 activation (Figure 3 and Figure 4).

CB1 is responsible for not only PEA-induced anti-nociceptive effects but also PEA-induced anti-inflammatory effects [40], which was further confirmed by using AM251, a CB1 receptor antagonist, in this study. The results showed that once CB1 was blocked, the upregulated expression of inflammatory cytokines *IL6* and *COX2* could not be effectively inhibited by treatment with the PEA-ENL in HaCaT cells (Figure 6). These results prove that PEA-ENL functions through CB1.

## 5. Conclusions

This study developed a PEA-loaded ENL and characterized its functionality. PEA was encapsulated into an ENL prepared with ceremide, cholesterol, and phosphatidylcholine with a nearly equal molar ratio as the stratum corneum lipids to better protect the skin barrier. The PEA-ENL showed considerably improved transdermal delivery capabilities and enhanced skin retention compared with free PEA. After PEA-ENL treatment, significant anti-nociceptive and anti-inflammatory effects were observed in HaCaT cells, mainly through regulation of CB1 and TRPV1. Therefore, this novel PEA-ENL is promising as an effective vehicle in the treatment of skin sensitivity and aging. Although the cost is relatively high, this ENL-based technology shows its potential for topical application in precision skincare, since the selection of molecules for specific pharmacological targets following precision transdermal delivery will be an effective treatment for skin problems and skin aging.

## Figures and Tables

**Table 1:** Human skin patch test results.

Group	Number of Participants	Observation Time	Number of Participants in Different Skin Reaction Scoring Levels for the Skin Patch Test				
			0	1	2	3	4
Control	16	0.5 h	16	0	0	0	0
		24 h	16	0	0	0	0
		48 h	16	0	0	0	0
PEA-ENL	16	0.5 h	16	0	0	0	0
		24 h	16	0	0	0	0
		48 h	16	0	0	0	0

## Data Availability

Available upon request.

## Supplementary Materials

The following supporting information can be downloaded at: https://www.mdpi.com/article/10.3390/pharmaceutics16070876/s1, Table S1. Integrated fluorescence intensity showing the in vitro transdermal delivery process. Figure S1. Particle size distribution of the PEA-ENL. Figure S2. Molecular docking sites and poses of CBIR-selective antagonist AM251 for CB1.
