# Supplementary material for: Palmitoylethanolamide-Incorporated Elastic Nano-Liposomes for Enhanced Transdermal Delivery and Anti-Inflammation
Source: Pharmaceutics. 2024 Jun 29;16(7):876. doi: 10.3390/pharmaceutics16070876 (PMC11280357; doi:10.3390/pharmaceutics16070876)
Supplement: Supplementary file 1 [file pharmaceutics-16-00876-s001.zip › pharmaceutics-3031497-supplementary.pdf]

Supplementary Materials: Palmitoylethanolamide-Incorporated Elastic Nano-Liposomes for Enhanced Transdermal Delivery and Anti-Inflammation

Chuanpeng Ren [1,*,†], Yanyun Ma [2,3,†], Yizhen Wang [1], Dan Luo [4], Yanhan Hong [4], Xinyuan Zhang [5], Hexiang Mei [1] and Wei Liu [6,*]

1 The Institute of Biocelline Precision Dermatology, Shanghai 200031, China
2 Human Phenome Institute, Fudan University, Shanghai, 201210, China
3 Institute for Six-Sector Economy, Fudan University, Shanghai 201203, China
4 Wuhan Bestcarrier Biotechnology Co., Ltd., Wuhan 430075, China
5 Shanghai Skinshield Clinical Testing and Technological Research Ltd., Shanghai 201210, China
6 National Engineering Research Center for Nanomedicine, Huazhong University of Science and Technology, Wuhan430074, China
* Correspondence: cp.ren@biocelline.com (C.R.); wliu@hust.edu.cn (W.L.)
† These authors contributed equally to this work.

Table S1. Integrated fluorescence intensity showing the *in vitro* transdermal delivery process. The porcine skin was treated with free RhoB or RhoB-loaded-ENL with equal RhoB concentration for up to 8 h. The fluorescence intensity was quantified by ImageJ software.

	**Time**	**Area**	**IntDen***
**Free RhoB**	2h	1233	123651
	4h	2390	145987
	8h	3687	296476
**RhoB-ENL**	2h	5485	317709.3
	4h	8861	516228.3
	8h	10806	627883.3

*IntDen: Integrated Density.

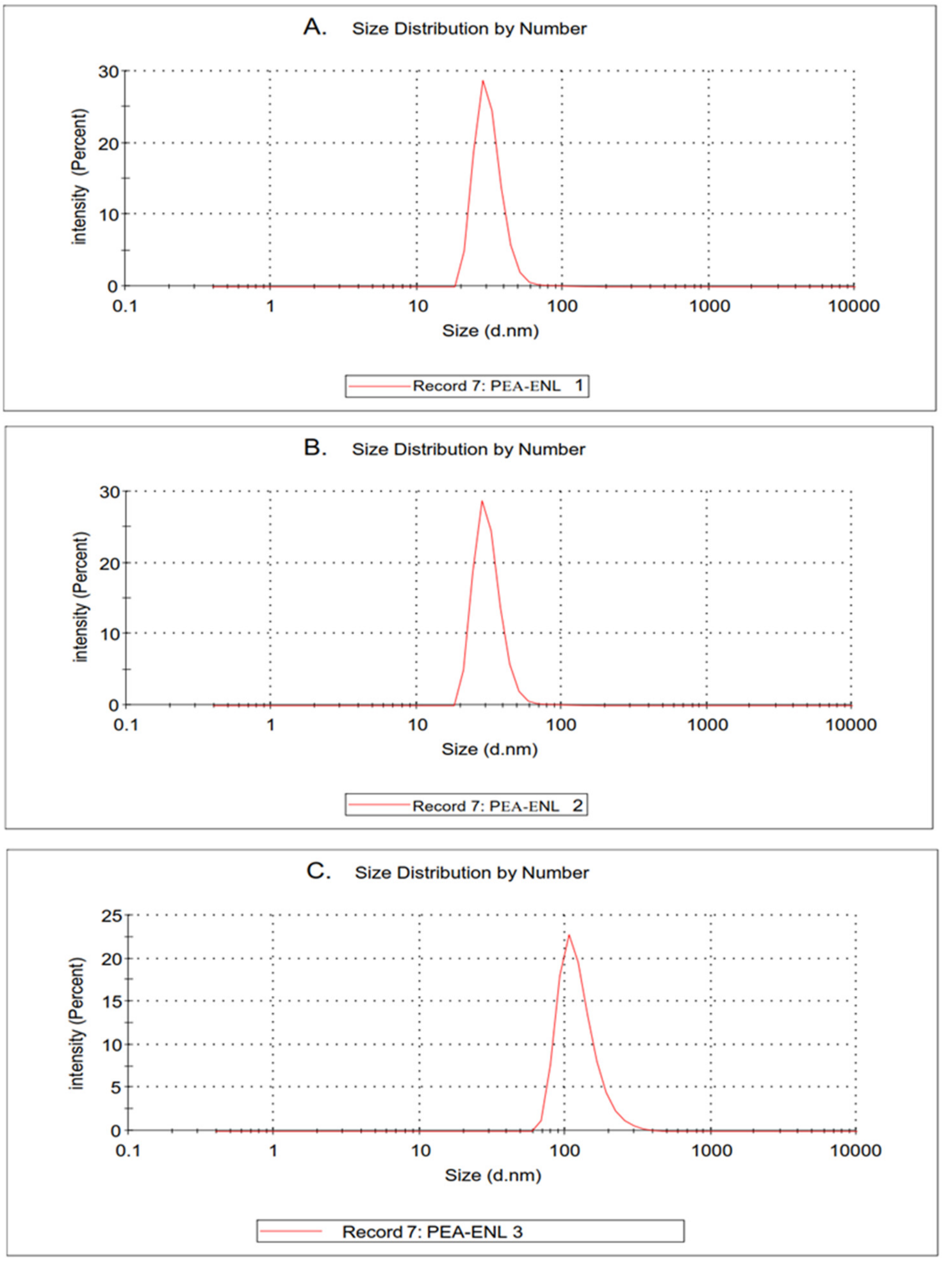

Figure S1. Particle size distribution of PEA-ENL measured by DLS.

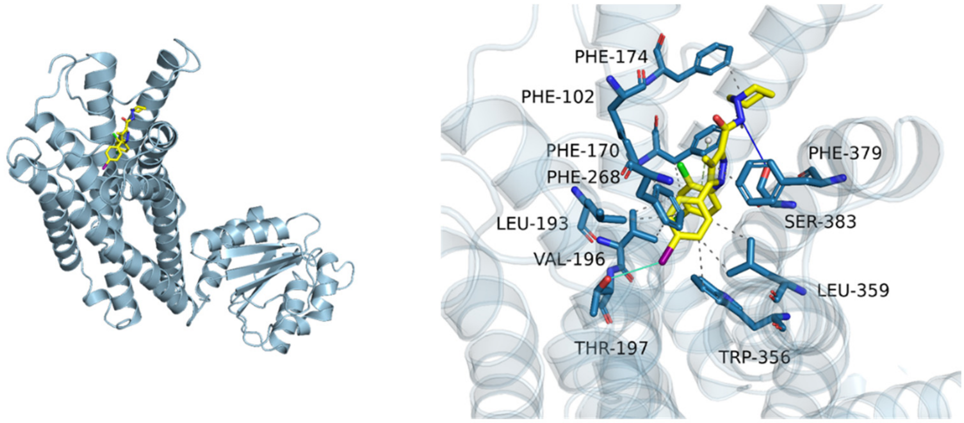

Figure S2. Molecular docking sites and poses of CBIR-selective antagonist AM251 for CB1 (PDB ID: 5TGZ). The docking score is -10.1 kcal /mol.
